# Evaluating how different balancing data techniques impact on prediction of premature birth using machine learning models

**DOI:** 10.1371/journal.pone.0316574

**Published:** 2025-04-02

**Authors:** Anna Beatriz Silva, Elisson da Silva Rocha, João Fausto Lorenzato, Patricia Takako Endo

**Affiliations:** Universidade de Pernambuco, Pernambuco, Brazil; VIT-AP Campus, INDIA

## Abstract

Premature birth can be defined as birth before 37 weeks of gestation, which is a significant global health issue, being the main cause for neonatal deaths. In this work, we evaluate machine learning models for predicting premature birth using Brazilian sociodemographic and obstetric data, focusing on the challenge of data imbalance, a common problem that can lead to biased predictions. We evaluate five data balancing techniques: Undersampling, Oversampling, and three Hybridsampling configurations where the minority class was increased by factors 2, 3, and 4. The machine learning models, including Decision Tree, Random Forest, and AdaBoost, are trained and evaluated on a dataset of over 483,000 cases. The use of the Hybridsampling approach resulted in an accuracy of 70%, a recall of 64%, and a precision of 74% in the Decision Tree model. Results show that Hybridsampling techniques significantly improves models’ performance compared to Undersampling and Oversampling, highlighting the importance of a proper data balancing in predictive models for preterm birth. The relevance of our work is particularly significant for the Brazilian Unified Health System (SUS). By improving the accuracy of premature birth predictions, our models could assist healthcare providers in identifying at-risk pregnancies earlier, allowing for timely interventions. This integration could enhance maternal and neonatal care, reduce the incidence of preterm births, and potentially decrease neonatal mortality, especially in underserved regions.

## 1 Introduction

According to the World Health Organization (WHO), newborns born before 37 weeks of gestation are considered premature or preterm [1]. Premature birth is classified based on the gestational period as follows: (a) extremely premature (less than 28 weeks), (b) very premature (28 to 31 weeks), and (c) moderate to late premature (32 to 37 weeks). The WHO estimates that in 2020, approximately 13.4 million babies were born prematurely worldwide, accounting for 9.9% of all live births [2,3]. Additionally, around one million children died from complications due to premature birth [4]. Many survivors face numerous disabilities, including visual and hearing problems. In Brazil, the rate of premature births is 12%, double that of European countries. The majority of occurrences are in the Northeast and Southeast regions of Brazil from 2011 to 2020 [2].

Artificial Intelligence (AI) models have become important in various contexts due to their ability to analyze vast amounts of data. In healthcare, AI models can assist in diagnosing diseases, predicting patient outcomes, and personalizing treatment plans. In the current literature, some works have presented solutions for the problem of predicting prematurity using AI models [5,6]. Our main contribution and focus in on the data imbalance in datasets used for predicting premature births.

It is crucial to address data balancing when using AI models, as highlighted by Jhonson et al. [7] and Nitesh et al. [8]. When AI models are employed for prediction problem, most data tend to favor the majority class (in our case, term births), resulting in a possible biased learning and poor representation of the minority class (premature births). This imbalance may lead to inaccurate and unreliable predictions for the minority class, impairing the effectiveness of AI models in practice.

Our work addresses the critical need to enhance the accuracy and reliability of AI models in predicting premature births. Given the high rates of prematurity and its severe consequences, it is imperative that prediction models adequately represent both majority and minority classes. By focusing on evaluating data balancing techniques on real sociodemographic and obstetric datasets from Brazil, this study aims to provide a more robust model for prematurity prediction, thereby contributing to more precise health interventions.

In this context, this work evaluates machine learning models for predicting premature birth using real data from SINASC (*Sistema de Informação sobre Nascidos Vivos*) that is a Brazilian open database with records related to sociodemographic and obstetric data related to pregnant women, available by the Brazilian Unified Health System (SUS). The focus is on the technical challenge of imbalanced data, where different balancing techniques are evaluated and discussed through a hybrid approach.

Therefore, our work stands out by focusing specifically on the impact of various data balancing techniques on the performance of machine learning models for predicting preterm birth. While previous research has explored preterm birth prediction using machine learning, there has been limited attention to systematically comparing different balancing methods, such as Undersampling, Oversampling, and Hybridsampling, in this context. Additionally, our study is distinctive in its exclusive use of clinical and sociodemographic data extracted from the Brazilian Unified Health System (SUS). Unlike other studies that may incorporate advanced biomarkers, genetic data, or specialized imaging, we focused on attributes readily available in SUS, ensuring our models are both practical and scalable in resource-constrained settings. This makes our approach particularly relevant for improving maternal and neonatal care in underserved regions, where access to sophisticated diagnostic tools is often limited.

The primary objectives of our research are threefold:

- To evaluate the performance of traditional tree-based machine learning models, such as Decision Trees, Random Forest, and AdaBoost, in predicting preterm birth using an imbalanced dataset;
- To systematically compare the effectiveness of different data balancing techniques, including Undersampling, Oversampling, and Hybridsampling, in improving the models’ ability to accurately predict preterm birth; and
- To highlight the practical implications of using sociodemographic and obstetric data for preterm birth prediction, particularly in low-resource settings, while identifying the limitations and opportunities for further improvement using more advanced models and techniques.

## 2 Theoretical foundation

In machine learning, particularly when using health data, imbalanced datasets can lead to biased and inaccurate predictions. Data imbalance can typically favor the majority class during model training, resulting in poor representation and unreliable predictions for the minority class [9].

In this section, we present the fundamental concepts for a better understanding of the methodology, including the description of AI models employed and the main evaluation metrics used.

### 2.1 Machine learning

Machine learning automates solutions for complex problems that are considered challenging to solve with conventional programming methods, offering a more effective alternative by learning from labeled datasets instead of requiring explicit designs. These algorithms generate models from data to make predictions, a process known as supervised learning [10].

In this study, we selected tree-based models as they are frequently used in similar works for binary classification prediction. The models include Decision Tree, Random Forest, and Adaboost.

#### 2.1.1 Decision tree

A Decision Tree is a popular machine learning model that can be used for both classification and regression tasks. It works by splitting the dataset into progressively smaller and more homogeneous subsets based on specific feature value tests. This splitting process creates a hierarchical, tree-like structure, where internal nodes represent decisions based on specific features, branches represent the outcomes of these decisions, and leaf nodes represent class labels in classification tasks or continuous values in regression tasks [11].

The construction of a Decision Tree involves selecting the best attribute at each stage using specific metrics. The best attribute is chosen to split the data, and this process continues recursively until the resulting subsets meet certain stopping criteria. The final structure is a tree where each path from the root node to a leaf represents a series of decisions leading to a classification or prediction [11].

Decision Trees are advantageous due to their straightforward interpretation, they do not require data normalization or feature standardization and can handle categorical data effectively. This could be applied in different fields. Decision Trees are powerful and intuitive tools in machine learning, providing a clear and (sometimes) interpretable approach for modeling decisions and making predictions based on data.

#### 2.1.2 Random forest

Random Forest is an ensemble learning algorithm that enhances predictive accuracy and robustness by leveraging Decision Trees. It generates a multitude of Decision Trees during training, with each tree built on a random subset of the data and a random subset of features [12]. This process, known as bootstrap aggregating or bagging, reduces overfitting and improves generalization. The strength of Random Forest lies in their ability to handle large datasets with high dimensionality and provide insights into feature importance.

#### 2.1.3 AdaBoost

AdaBoost is a boosting algorithm that enhances the performance of weak classifiers by combining them into a single strong classifier. The process involves training multiple weak learners sequentially, where each new learner focuses on the mistakes made by the previous ones. The algorithm adjusts the weights of the training instances, increasing the weight of misclassified instances so that subsequent classifiers pay more attention to them. This iterative process continues until a predefined number of classifiers are trained or the model achieves a specified accuracy. AdaBoost is effective for improving the accuracy of models with weak predictive power and is robust to overfitting when used with proper regularization [13].

### 2.2 Metrics

The evaluation metrics were established according to standards recognized in the current literature. The confusion matrix is commonly used to evaluate machine learning classification models. The matrix provides a detailed view of model performance and compares it with actual results. The matrix provides a table that compares predicted classes with actual classes and includes the following elements:

- True Positive (TP): Correctly predicted positive class results.
- False Negative (FN): Incorrectly predicted negative class when ti should have been positive.
- False Positive (FP): Incorrectly predicted positive class result when it should been negative.
- True Negative (TN): Correctly predicted negative class results.

From the confusion matrix, other metrics can be calculated to assess a model performance, such as accuracy, recall, precision, specificity and F1-Score.

#### 2.2.1 Accuracy

Accuracy is defined as the proportion of TP and TN among all the tested data [14], as shown in Eq 1:


$$\begin{array}{ccc} accuracy=\frac{TP+TN}{TP+TN+FP+FN} & & \end{array}$$ (1)

#### 2.2.2 Recall

Recall (also known as the true positive rate) measures a model’s ability to correctly identify the positive class. In health-related problems, recall is important as it measures a model’s ability to correctly detect positive cases of a disease, as show in Eq 2:


$$\begin{array}{ccc} recall=\frac{TP}{TP+FN} & & \end{array}$$ (2)

#### 2.2.3 Precision

Precision measures the proportion of cases classified as positive by a model that are actually positive in relation to all the true cases classified by a model, as shown in Eq 3:


$$\begin{array}{ccc} precision=\frac{TP}{TP+FP} & & \end{array}$$ (3)

#### 2.2.4 Specificity

Specificity, which identifies negative instances, helps in understanding how well a model is detecting true negative cases, as per Eq 4:


$$\begin{array}{ccc} specificity=\frac{TN}{TN+FP} & & \end{array}$$ (4)

#### 2.2.5 F1-Score

F1-Score, a metric that combines precision and recall into a single measure, provides a more balanced view of a model’s classification performance, being calculated as presented in Eq 5:


$$\begin{array}{ccc} F1-score=2\times \frac{precision\times sensitivity}{precision+sensitivity} & & \end{array}$$ (5)

### 2.3 Balancing techniques

Data balancing is a crucial stage in machine learning model training, especially in scenarios involving imbalanced datasets. A dataset is considered imbalanced when the number of samples in one class is lower than other classes. This imbalance could negatively impact the model performance since the models may favor the majority classes. In this context, there are different techniques to address this problem. This study will use Undersampling, Oversampling, and Hybridsampling techniques.

#### 2.3.1 Undersampling

Undersampling is a technique used to balance class distributions by reducing the number of instances in the majority class to match the same amount in the minority class [15,16]. One common approach to using the Undersampling is through Random Undersampling, where a subset with the majority class is randomly selected, this selection ensures that the model does not overfit to a specific pattern in the majority class.

However, a potential disadvantage of Undersampling is that it can lead to the loss of valuable information because of the discarded majority class data. This approach between balancing the classes and retaining information must be carefully analyzed, considering that an excessive reduction of majority class data might compromise the model’s ability to learn important patterns. Therefore, selecting the appropriate Undersampling technique and evaluating its effect on model performance is crucial to ensure that the model retains sufficient information for performance in the predictions.

#### 2.3.2 Oversampling

Oversampling involves increasing the data of the minority class so that it matches or resembles the majority class [15,17].

This technique is used for imbalanced datasets, where the minority class has too few instances to train a model. One straightforward method of Oversampling is random duplication, named Random Oversampling, where existing instances of the minority class are duplicated to increase their representation in the dataset. While this can help balance the classes, it may also lead to overfitting, as the model might learn to recognize duplicated instances rather than general patterns [18].

#### 2.3.3 Hybridsampling

Hybridsampling is an approach that involves both the creation and removal of existing data in the model, using Oversampling and Undersampling simultaneously. This technique combines the two methods to reduce the amount of data in the majority class and increase the amount of data in the minority class through the creation of synthetic data [19].

To reduce the data, the Random Undersampling algorithm is used. Then, to create synthetic data in the minority class, we used SMOTE (Synthetic Minority Over-sampling Technique). SMOTE is an algorithm that finds examples of minority classes that are close to each other in a given search space by calculating the distances between instances using some distance metric. For each example in the minority class, SMOTE randomly selects one of its close neighbors (k-neighbors) and creates a synthetic example along the line that connects them [20]. Finally, the synthetic examples are added to the original dataset, thus increasing the representation of the minority class.

### 2.4 Hyperparameter optimization

Hyperparameters are variables configured before training a machine learning model. The appropriate choice of hyperparameters can improve model’s performance.

In this work, to define the best hyperparameters for each model, a technique called Grid Search was used. The technique finds the best combination of hyperparameters for a machine learning model. Grid Search used accuracy as the evaluation metric for the combination of these hyperparameters, and each scenario has its own set of hyperparameters [21,22]. This happens because, when running each model before the training process, a grid is generated and then the model is trained with the best parameters found, resulting in a different set of parameters for each model and hyperparameter combinations.

### 2.5 Related work

Recent studies in the literature have presented AI solutions to address the issue of preterm birth. For instance, Yerlikaya et al. [23] present an AI model for predicting premature birth using sociodemographic and clinical data from obstetric screenings of pregnant women. Using this data, the study highlights the linear relationships between the main attributes of the database, demonstrating rinks linked to clinical factors such as placental compromise and maternal factors such as the Body Mass Index (BMI) of the pregnant woman.

Similarly, Arabi [24] presents a model for predicting premature birth in nulliparous women. Using a database from the Better Outcomes Registry and Network (BORN) from Ontario, Canada. The study identifies predictors related to premature birth using logistic regression and the most severe complications associated with pregnancy, predicting the main factors according to the gestational trimester.

The need of data balancing in predicting preterm birth is also a focus in literature. Włodarczyk et al. [25] systematically review the applicability of AI models for predicting preterm birth. In their study, Włodarczyk et al. emphasize the importance of imbalanced data and the impacts this type of dataset can have on models. Additionally, they highlight the most efficient balancing techniques for handling such data.

On the other hand, the work by Zhang et al. [26] present prediction models from a small data sample without performing balancing. Despite this, Zhang’ models present good performance, demonstrating that the models are efficient for clinical data related to preterm birth.

Table 1 summarizes these approaches, highlighting the main attributes analyzed, sample sizes, models employed, and achieved performance metrics. This comparison demonstrates how the careful selection of variables, applied methodology, and data balancing can significantly impact the accuracy of predictive models in studies focused on preterm birth prediction.

**Table 1:** Summary of current literature works for preterm birth prediction using AI.

Authors	Attributes	Registers	Models	Results (AUC)
Yerlikaya et al. [23]	Age, Wt, Ht, Race, Conception, Smoker, HTN, APS/SLE, DM, Nulliparous, Miscarriage, Stillbirth, SGA, Interpregnancy interval	113,415	Univariable and multivariable logistic regression analysis	0.642
Arabi et al. [24]	Age, Ht, Pre-pregnancy BMI, GWG (1st trim), Income, Educ, Race, Immig. status, Smoking, Alcohol, Folic acid, Med cond, Mental cond, Conception (e.g., spont/IVF), PAPP-A, Free B-hCG, Nuchal trans, Inhibin A, Estriol, hCG, AFP, Hypertens, GDM, Infections, Med exposure, Fetus sex, Complications	112,963	Logistic Reg., ANN, RF, DT	1st trim: 0.53–0.60 2nd trim: 0.58–0.80
Włodarczyk et al. [25]	Age, BMI, Prev preterm, Smoking, Alcohol, HTN, DM (incl. GDM), Uterine anomalies, Cervical length, Cervix consis. index, Cervical angle, RMS (EHG), Peak freq, Median freq, Samp entropy, Burst dur (EMG), Burst freq, Total act.	EHR: Up to 15M EHG: 300 TVS: 350 EMG: 87–185	Log Reg., SVM, KNN, DT, RF, NN, Grad Boost, NB, AdaBoost, Bagging	0.69–0.96
Zhang et al. [26]	Parity, Neck loops, Age, Abort hist, Placenta previa, PPROM, HTN, DM, Anemia, Mult. preg., Fetal position, Prenat. hemorrhage, Uterine abn, Scar uterus, Fibroids, Fetal distress, S. agalactiae, AF traits, Abn. AF vol, Assist. preg., Fetal Wt abn.	5,411	Log Reg., DT, NB, SVM, AdaBoost	0.945–0.953

Source: Table by the author.

Our study extends the existing work by focusing on the evaluation of balancing techniques alongside machine learning training to further improve the models’ performance used to predict preterm births.

## 3 Materials and method

### 3.1 Dataset

The dataset used in this study is from the SINASC, containing 526.368 records and 61 attributes related to pregnant women, from 2018 to 2021, of the state of Pernambuco, Brazil. The SINASC is a system implemented by the Brazilian federal government in the 1990s, with the purpose of collecting data on all live births in the national territory. The system makes it possible to provide information on birth rates for all levels of the Brazilian health system, as well as the development of relevant indicators in the strategic planning of management to support the planning of actions, activities, public policies and programs aimed at health. The (preprocessed) dataset used in this work is available in Mendeley Data at the following url: https://data.mendeley.com/datasets/z3ychcthm2/3.

**Fig 1:**
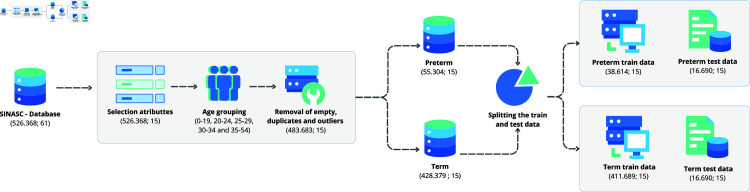
Preprocessing steps performed to build the final dataset.

The data preprocessing comprised some steps, as described in Fig 1: identification of relevant attributes, grouping of age ranges, and removal of empty data, duplicates, and outliers. Considering that our goals is to train models for predicting premature birth, we consider two distinct target classes of this dataset: Preterm and Term.

The first step of processing begins with identifying attributes that are not relevant to the scope of our work, which were excluded. This involved a manual analysis to remove attributes categorized as system attributes, medical or hospital data related to the birth, attributes related to the birth of the fetus, and maternal attributes. On the other hand, attributes based on the current literature considered highly important in preterm birth occurrences were utilized [27]: mother’s age during pregnancy, history of preterm birth, multiple pregnancies (twins, triplets, or more), chronic diseases, infections, genetic influences, nutritional factors, and the woman’s lifestyle.

The next step involved analyzing the mother’s age distribution due to the wide range of values for this attribute. To establish the age ranges, we used averages from previous studies. For this, previous studies were analyzed to define this age scale for similar health problems. According to Fuchs et al. [28], Waldenström et al. [29], and Nathalie et al. [30], the age range distribution remains very similar, maintaining maternal age patterns related to the risk of premature birth. This highlights not only the importance of considering older ages but also factors such as young age during pregnancy. For Schempf et al. [31], this pattern differs in the dispersion of these scales, with a smaller distance between ages, resulting in very small groupings, as presented in Table 2.

**Table 2:** Age group distribution according to the literature.

Author	Age group					
Fuchs et al. [28]	< 20	20–24	25–29	30–34	35–39	≥ 40
Waldenstrom et al. [29]	< 20	20–24	30–34	35–39		≥ 40
Auger et al. [30]	< 20	20–24	25–29	30–34	35–39	≥ 40
Schempf et al. [31]	< 17	18–19	20–24	25–29	30–34	≥ 35

Source: Table by the author.

This approach allowed for more precise and representative segmentation, considering patterns accepted and used in earlier research. The age ranges were defined to reflect different stages, resulting in intervals of 0–19, 20–24, 25–29, 30–34, and 35–54. Empty or duplicate records were excluded from the dataset, ensuring greater integrity and consistency of the data used.

It is worth noting that the dataset used in this study contains only sociodemographic attributes and the obstetric history of the mother. Information regarding the health history of the pregnant woman is not available. Therefore, understanding the existing characteristics in the dataset was the main criterion applied for the final selection of the attributes used to train our models. At the end of this first processing step, 15 attributes remained for our model training, as show in Table 3. The excluded attributes can be see in Table 4.

**Table 3:** Selected attributes.

#	Attribute	Description	Type
1	ESTCIVMAE	Marital status.	Categorical
2	QTDFILVIVO	Number of living children.	Numeric
3	QTDFILMORT	Number of fetal losses and miscarriages.	Numeric
4	GESTACAO	Weeks of pregnancy.	Categorical
5	GRAVIDEZ	Type of pregnancy.	Categorical
6	SEXO	Sex.	Categorical
7	RACACORMAE	Mother’s race/color.	Categorical
8	QTDGESTANT	Number of previous pregnancies.	Numeric
9	QTDPARTNOR	Number of vaginal births.	Numeric
10	QTDPARTCES	Number of cesarean deliveries.	Numeric
11	ESCMAEAGR1	2010 aggregate education.	Categorical
12	MESPRENAT	Month of pregnancy in which prenatal began.	Categorical
13	PARIDADE	First pregnancy or multiparous.	Categorical
14	IDADEMAE	Mother’s age in years.	Numeric
15	CLASSE	Target.	Binary

Source: Table by the author.

**Table 4:** Attributes removed after preprocessing.

Attributes	Description
CODOCUPMAE	Mother’s occupation.
ESCMAE	Education, years of study completed.
ESCMAE2010	Education 2010.
SERIESCMAE	Mother’s school series.
SEMAGESTAC	Number of weeks of gestation.
IDADEPAI	Father’s age.
DTNASCMAE	Mother’s date of birth.
STDNEPIDEM	Epidemiological DO Status.
STDNNOVA	New DO status.
TPAPRESENT	Month of pregnancy in which prenatal care began.
STTRABPART	Induced labor?
TPMETESTIM	Method used.
IDANOMAL	Congenital anomaly.
CONSULTAS	Number of prenatal consultations.
CODANOMAL	Congenital malformation or anomaly code chromosomal.
CODESTAB	Code of the health establishment where the birth took place.
CODMUNNASC	Code of the municipality of occurrence.
LOCNASC	Place of birth.
CODMUNRES	Mother’s municipality of residence.
DTNASC	Date of birth.
HORANASC	Time of birth.
PESO	Birth weight, in grams.
APGAR1	Apgar in the first minute (00 to 10).
APGAR5	Apgar in the fifth minute (00 to 10).
DTCADASTRO	Date of registration of the DN in the system.
NUMEROLOTE	Lot number.
DTRECEBIM	Date of receipt at central level, date of last registry update.
DIFDATA	Time between death and receipt of DO.
DTRECORIGA	Create DTRECORIGA field and copy DTRECORIG values.
NATURALMAE	The country of birth code.
CODMUNNATU	Code of the mother’s municipality of birth.
CODUFNATU	UF code of mother’s place of birth.
DTULTMENST	Date of last menstrual period (LMP).
CONSPRENAT	Number of prenatal consultations.
STCESPARTO	Did the cesarean section occur before labor started?
TPNASCASSI	Birth was attended by?
TPFUNCRESP	Type of role of the person responsible for filling it out.
TPDOCRESP	Type of document of the person responsible.
DTDECLARAC	Declaration date.
CODPAISRES	Country of residence code.
TPROBSON	Robson Group Code, generated by the system.
KOTELCHUCK	Prenatal care index measures pregnancy healthcare quality.
CONTADOR	Record identification number.
ORIGEM	Source Database.
RACACOR	Race/Color.

Source: Table by the author.

Following this data treatment, we divided the dataset considering the classes Term and Preterm: Term includes all data where the gestational period is greater than 37 weeks, while Preterm encompasses all data with a gestational period of 37 weeks or less. The Term class represents 88.97% of the total dataset.

Before performing data balancing, a strategy was implemented to ensure a realistic representation of the data for model testing. Therefore, 30% of the total data from the minority class (Preterm) and an equal amount from the majority class (Term), equivalent to 16.690 records, were separated. This approach avoids data leakage issue when training machine learning models [32], and ensures the validity of tests for different scenarios.

### 3.2 Experiments

**Fig 2:**
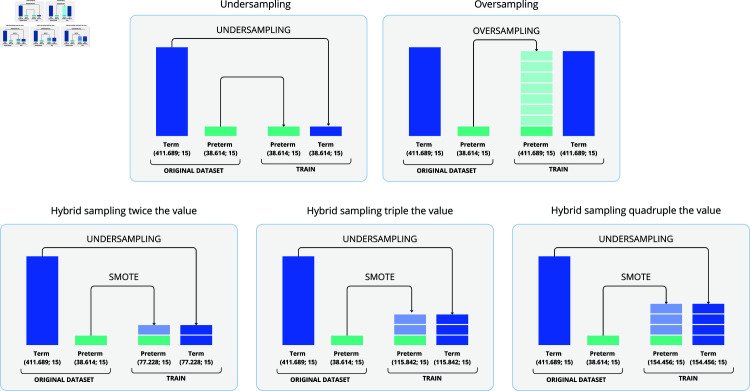
Sampling scenarios used in our experiments.

The experiments consisted of creating distinct scenarios using different datasets (according to data balancing technique) to be used as input for different machine learning models: Decision Tree, Random Forest and AdaBoost. Fig 2 shows the sampling scenarios.

**Fig 3:**
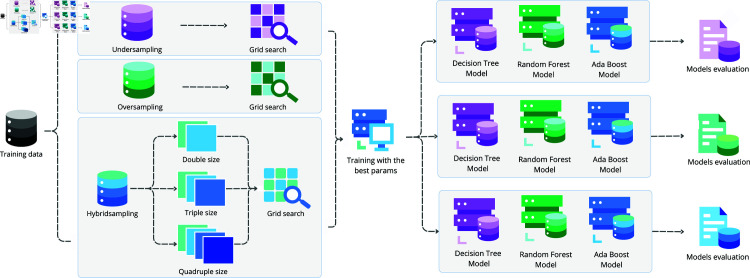
Overview for handling imbalanced data and training machine learning models.

For selecting models’ hyperparameters, Grid Search was used as described in Table 5, and accuracy was selected as the evaluation metric for the combination of these hyperparameters, and each scenario has its own set of hyperparameters (see Fig 3).

**Table 5:** Grid search hyperparameters.

Models	Hyperparameters
Decision Tree	SPLIT_CRITERION: [‘gini’, ‘entropy’]
	MAX_DEPTH: [None, 5, 10, 15]
	MIN_SAMPLES_SPLIT: [2, 5, 10, 15]
	MIN_SAMPLES_LEAF: [1, 2, 4, 10]
Random Forest	N_TREES: [25, 50, 100]
	MAX_DEPTH: [None, 10, 20]
	MIN_SAMPLES_SPLIT: [2, 5, 10]
	MIN_SAMPLES_LEAF: [1, 2, 4]
AdaBoost	N_ESTIMATORS: [25, 50, 100]
	LEARNING_RATE: [1.0, 2.0, 3.0]
	LOSS_FUNCTION: [‘SAMME’, ‘SAMME.R’]

For the Decision Tree model, the hyperparameters evaluated were the SPLIT_CRITERION, which specifies the function used to measure the splits. Two options were selected for this hyperparameter: Gini and Entropy. The Gini criterion minimizes the Gini impurity, while Entropy is based on information gain. Both influence the modification of the node’s purity. The MAX_DEPTH defines the maximum depth of the three, balancing the bias-variance trade-off. The MIN_SAMPLES_SPLIT represents the minimum number of samples required to split a node. The MIN_SAMPLES_LEAF determines the minimum number of samples required in each leaf, controlling the leaf size and influencing the stability and variance of the tree.

For the Random Forest model, the hyperparameters included the N_TREES that defines the number of trees in the forest; MAX_DEPTH, that limits the depth of each tree, mitigating the risk of overfitting; MIN_SAMPLES_SPLIT, similar to the Decision Tree model, controls the minimum number of samples required to split a node, affecting the granularity of the splits; and MIN_SAMPLES_LEAF, that defines the minimum number of samples required in each leaf, influencing the bias and variance of the individual tree in the ensemble.

For the AdaBoost model, we used a Decision Tree classifier with a MAXIMUN DEPTH of 1, known as a decision stump. The hyperparameters were the N_ESTIMATORS, which defines the number of estimators or boosting rounds; LEARNING_RATE regulates the contribution of each classifier in the sequence; and the LOSS_FUNCTION where SAMME uses the exponential loss function and SAMME.R is based on the logarithmic loss, influencing the weight adjustment of misclassified samples in each iteration of the ensemble.

This hyperparameter configuration was designed to adequately explore the solution space, seeking the ideal balance between fitting capacity and model generalization. This study’s model evaluation metrics are accuracy, recall, F1-score, precision, and specificity.

## 4 Results

### 4.1 Undersampling

This experiment consisted exclusively of applying the Undersampling balancing technique. The number of records from the majority class was reduced to equal the quantity of data from the minority class, totaling 38.614 records for each class. The best hyperparameters selected for this experiment can be seen in Table 6.

**Table 6:** Best hyperparameters when using undersampling.

Models	Hyperparameters
Decision Tree	SPLIT_CRITERION: ‘entropy’
	MAX_DEPTH: 10
	MIN_SAMPLES_SPLIT: 15
	MIN_SAMPLES_LEAF: 1
Random Forest	N_TREES: 100
	MAX_DEPTH: 10
	MIN_SAMPLES_SPLIT: 2
	MIN_SAMPLES_LEAF: 2
AdaBoost	N_ESTIMATORS: 100
	LEARNING_RATE: 1.0
	LOSS_FUNCTION: ‘SAMME.R’

**Fig 4:**
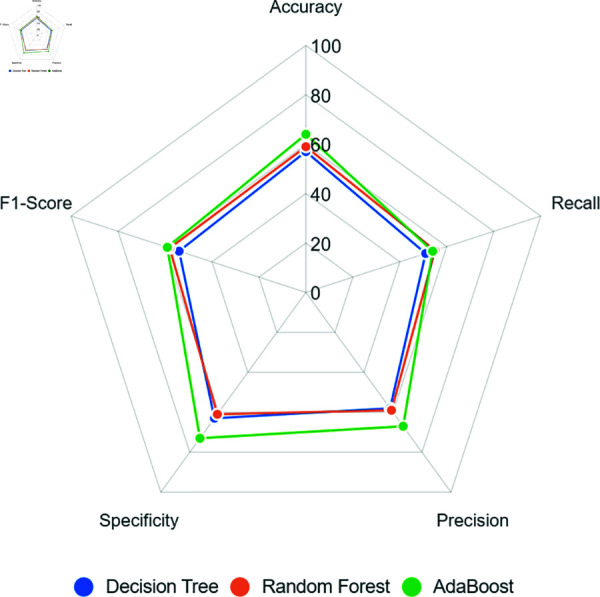
Models’ performance when using Undersampling to balance the training dataset.

Fig 4 presents a radar chart that distributes the evaluation metrics values on a scale of 0 to 100. Results obtained with Undersampling show relatively low performance for all models. Especially regarding recall, crucial for assessing the model’s ability to correctly detect records classified as positive, all models obtained values lower than 55%, indicating a very low learning performance.

Despite this, models’ specificity had a slightly better performance, reaching about 70% as observed in the AdaBoost model. These results imply the need to review the Undersampling balancing and explore other techniques to correct the data discrepancy, as shown in next sections.

### 4.2 Oversampling

In the same way, the use of Oversampling to balance the dataset was explored. Each class was composed of 458.657 records, with the Preterm class increased by 403,879 new synthetic data points. The best hyperparameters selected for this experiment is shown in Table 7.

**Table 7:** Best hyperparameters when using oversampling.

Models	Hyperparameters
Decision Tree	SPLIT_CRITERION: ‘entropy’
	MAX_DEPTH: ‘None’
	MIN_SAMPLES_SPLIT: 2
	MIN_SAMPLES_LEAF: 1
Random Forest	N_TREES: 100
	MAX_DEPTH: 10
	MIN_SAMPLES_SPLIT: 2
	MIN_SAMPLES_LEAF: 2
AdaBoost	N_ESTIMATORS: 25
	LEARNING_RATE: 1.0
	LOSS_FUNCTION: ‘SAMME.R’

**Fig 5:**
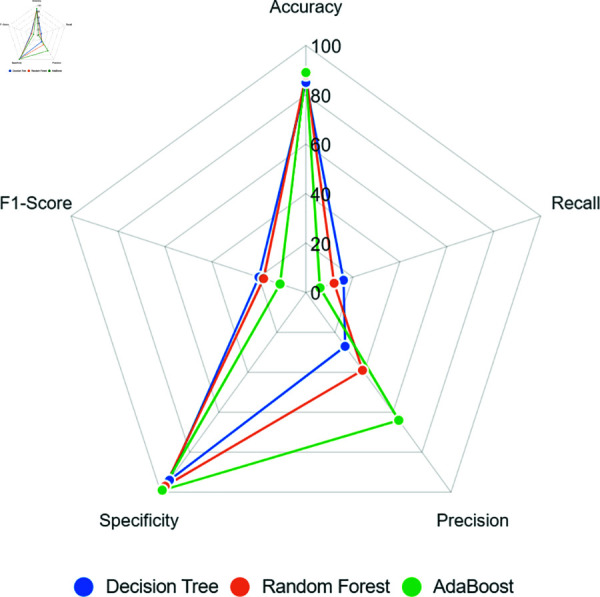
Models’ performance when using oversampling to balance the training dataset.

Fig 5 presents the models’ performance and despite all models obtain an accuracy above 85%, other metrics such as recall was below 15% in all models, indicating a limited positive detection rate. In contrast, specificity exceeded 90%, reaching up to 99%, demonstrating a strong detection performance for the Term class.

This disparity in models’ performance not only demonstrates the possible bias but also highlights the inefficacy of *Random Oversampling* when using our dataset in this study.

### 4.3 Hybridsampling

For the application of the Hybridsampling technique, synthetic data were created based on data from the minority class multiplied by double, triple and quadruple size of its original value.

#### 4.3.1 Hybridsampling double size

The minority class was increased to twice of its original size, while the majority class was reduced to match the same data quantity as the minority class, resulting in 77.228 samples for each class. After selecting the best hyperparameters, presented in Table 8, the models were trained with these hyperparameters, and evaluated.

**Table 8:** Best hyperparameters when using Hybridsampling double size.

Models	Hyperparameters
Decision Tree	SPLIT_CRITERION: ‘gini’
	MAX_DEPTH: 10
	MIN_SAMPLES_SPLIT: 15
	MIN_SAMPLES_LEAF: 1
Random Forest	N_TREES: 100
	MAX_DEPTH: 20
	MIN_SAMPLES_SPLIT: 10
	MIN_SAMPLES_LEAF: 1
AdaBoost	N_ESTIMATORS: 100
	LEARNING_RATE: 1.0
	LOSS_FUNCTION: ‘SAMME.R’

Table notes: The table lists the models and their corresponding hyperparameters used for Grid Search.

**Fig 6:**
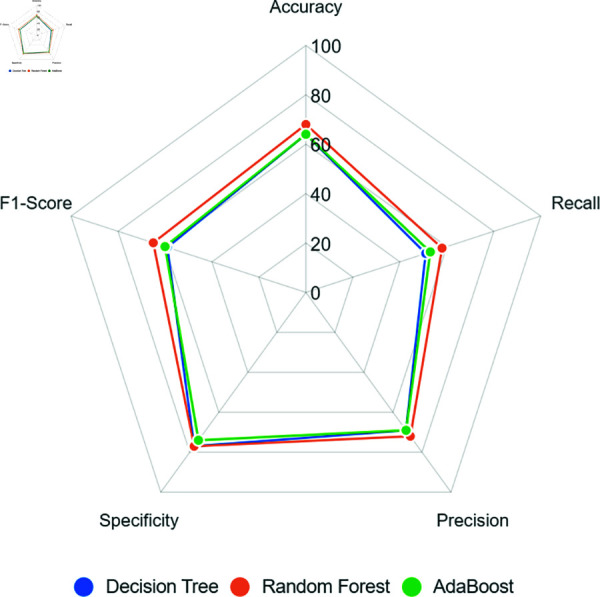
Models’ performance when using Hybridsampling double size to balance the training dataset.

Fig 6 shows that all models present a similar accuracy, with the Decision Tree and Adaboost reaching 64%. Random Forest stood out, reaching 58% of recall, the biggest value on this metric, despite this indicating a low ability of the model to correctly identify the minority class, highlighting a weak learning.

On the order hand, precision and specificity metrics showed better results than previous scenarios. For example, Adaboost achieved 67% of precision and 73% of specificity, suggesting a greater learning of the majority class.

#### 4.3.2 Hybridsampling triple size

In this case, we increased the minority class on triple size, compared to its original size, totaling 115.842 samples for the Preterm class. Then, the Term class was reduced to have the same amount of data as the Preterm class. For the model training, the selection of the best hyperparameters for each model was also performed, as shown in Table 9.

**Table 9:** Best hyperparameters when using Hybridsampling triple size.

Models	Hyperparameters
Decision Tree	SPLIT_CRITERION: ‘entropy’
	MAX_DEPTH: ‘None’
	MIN_SAMPLES_SPLIT: 2
	MIN_SAMPLES_LEAF: 1
Random Forest	N_TREES: 100
	MAX_DEPTH: ‘None’
	MIN_SAMPLES_SPLIT: 5
	MIN_SAMPLES_LEAF: 2
AdaBoost	N_ESTIMATORS: 50
	LEARNING_RATE: 1.0
	LOSS_FUNCTION: ‘SAMME.R’

Table notes: The table lists the models and their corresponding hyperparameters used for Grid Search.

**Fig 7:**
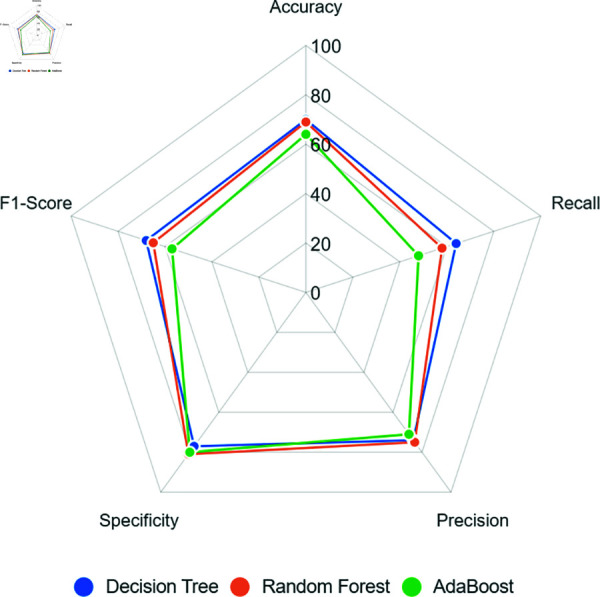
Hybridsampling triple size models performance.

Fig 7 shows that models’ performance for this scenario was better than the scenario with twice the values of the minority class, with the Decision Tree reaching up to 70% of accuracy. However, recall showed a significant difference from the previous scenario, where the Decision Tree obtained the highest value, with 64%; the Random Forest maintained a performance of 58%, and the AdaBoost was less than 50%, demonstrating a weak learning in this case.

The decision tree-based models still maintained satisfactory evaluation metrics, where precision and specificity in both surpassed 74%, demonstrating an efficiency learning for the majority class. Decision Tree and Random Forest models reaching 68% and 65% of F1-score, respectively. Indeed, these performance represent an improvement in the learning context for the minority class, demonstrating that the creation of new (synthetic) data, or the use of few data, impacts the learning of these models.

#### 4.3.3 Hybridsampling quadruple size

The Hybridsampling was characterized by a fourfold increase in data creation compared to the original Preterm class data, while the majority class was reduced to the same data amount as the minority class, totaling 154.456 records. The best hyperparameters were selected for model training, as shown in Table 10.

**Table 10:** Best hyperparameters when using Hybridsampling quadruple size.

Models	Hyperparameters
Decision Tree	SPLIT_CRITERION: ‘entropy’
	MAX_DEPTH: ‘None’
	MIN_SAMPLES_SPLIT: 5
	MIN_SAMPLES_LEAF: 1
Random Forest	N_TREES: 100
	MAX_DEPTH: ‘None’
	MIN_SAMPLES_SPLIT: 10
	MIN_SAMPLES_LEAF: 1
AdaBoost	N_ESTIMATORS: 50
	LEARNING_RATE: 1.0
	LOSS_FUNCTION: ‘SAMME.R’

Table notes: The table lists the models and their corresponding hyperparameters used for Grid Search.

**Fig 8:**
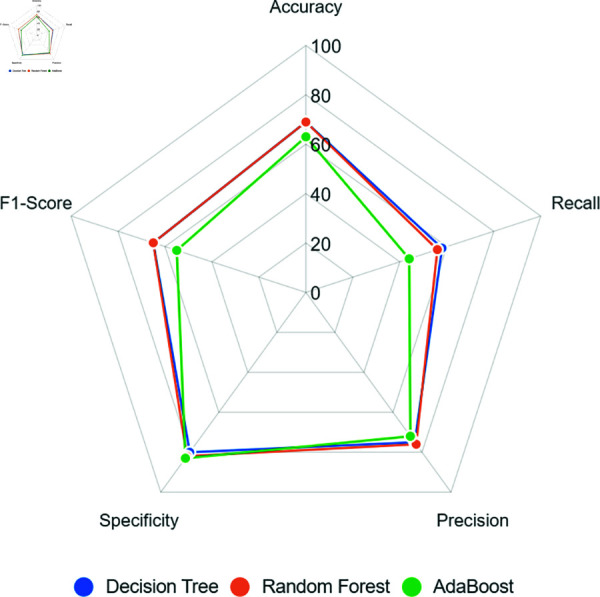
Models’ performance when using Hybridsampling quadruple size to balance the training dataset.

Fig 8 shows that models’ performance were lower compared to the experiment with Hybridsampling triple size. Decision Tree and Random Forest achieved 69% of accuracy, while Adaboost model reached only 63% of accuracy, the lowest recorded for Adaboost in this scenario. Recall across all models was also below 58% for Decision Tree, 56% for Random Forest, and 44% for Adaboost. These low values indicate weak learning for all models regarding the minority class, especially for Adaboost. This might be due to Adaboost’s learning approach, as it prioritizes incorrectly classified samples in each subsequent iteration, aiming to resolve the most challenging ones. This approach may enhance overall performance but also makes the model more sensitive to outliers or noise. Using weak classifiers, such as simple Decision Trees, as a base, can result in less robust initial performance, especially on complex datasets.

Regarding precision and specificity, models achieved better performance, ranging from 72% to 76% and 80% to 83%, respectively, indicating efficiency model assertiveness for false positives. The F1-score remained at 65% for Decision Tree and Random Forest.

Among the three scenarios analysed in the Hybridsampling technique, the Hybridsampling triple size achieved better metrics compared to the other scenarios. This is particularly evident in the Decision Tree’s accuracy, which reached 70%, and recall, which was 64%, the highest values obtained in the experiments. Furthermore, the Hybridsampling triple size scenario also achieved the best F1-score, 68%, along with satisfactory specificity and precision values of 77% and 74%, respectively. These metrics demonstrate a better balance in the models for learning each class, maintaining satisfactory metrics, and showing greater efficiency in understanding both classes.

We can assert that, based on the technique of creating synthetic data, there is a significant contribution this type of balancing can make to model learning. However, despite this contribution, it is evident that each dataset will differ from others. There will be cases where generating a certain amount of data will be beneficial only up to a certain point. This can be observed in the experiment Hybridsampling quadruple size, where, after a growing curve in evaluation metric values in the double and triple size scenarios, all models and metrics showed a decline in performance. This indicates that the excessive creation of synthetic data proved detrimental to the models in question.

From this perspective, the scenario Hybridsampling triple size was selected as the best-performing scenario among the Hybridsampling scenarios.

## 5 Discussions

Based on the results presented, we observed that data balancing techniques directly impact on the models’ learning and performance. The undersampling technique produces a substantial reduction in the training data, which consequently led to a degradation in the model learning. On the other hand, caution is necessary when generating synthetic data to balance the classes, as it can influence model outcomes both positively and negatively, impacting it overall performance.

In the Oversampling experiment, models exhibited the worst performance, with low recall and F1-score, and it also showed a bias towards the majority class. Despite high accuracy across all tested models, the Oversampling technique proved inefficient in addressing the problem of our study.

When the Undersampling technique was applied, the models demonstrated somewhat improved performance, exhibiting better metrics compared to those achieved with the Oversampling technique. However, the recall values were persistently low, suggesting that the models struggled with accurately identifying instances of the minority class.

When the Hybridsampling technique was employed, the models exhibited the best performance across all tested scenarios. Particularly, tripling the original dataset volume resulted in the highest metrics compared to other experiments. This underscores the potential necessity for models to access more extensive data from minority classes to enhance their understanding. Given the healthcare context, instances of negative outcomes (i.e., sick patients) are less frequent than positive ones (healthy individuals), the strategic augmentation of data through balancing techniques can be helpful for developing effective predictive models.

### 5.1 Limitations

Despite the promising results obtained in this study, several limitations should be acknowledged. The dataset used in our analysis spans from 2018 to 2021, which, while providing a robust foundation for model development and evaluation, may not fully reflect recent trends or shifts in risk factors and healthcare practices up to 2024. The challenge of incorporating more recent data lies in the delays associated with data collection, processing, and public release, which can impact the relevance and timeliness of our predictions. Regarding accuracy, while 70% might appear modest in some contexts, it is important to consider the inherent complexity and variability of preterm birth prediction using only clinical data. The reported accuracy, coupled with a recall of 64% and precision of 74%, demonstrates a balanced performance, effectively identifying at-risk pregnancies while minimizing false alarms.

Another limitation is the lack of comprehensive clinical attributes in the SINASC dataset. Our study relied primarily on sociodemographic and obstetric information without access to other health indicators, such as laboratory results, genetic factors, or maternal health history. This absence of detailed clinical data may restrict the predictive accuracy of our models, as risk factors for preterm birth are not captured in the available dataset. At the same time, however, this reliance on readily available attributes allows our models to be applied in settings with limited resources, such as locations that lack access to laboratory tests or advanced clinical diagnostics. This makes our approach potentially valuable for underserved areas, where basic sociodemographic and obstetric data are more accessible and could still aid in identifying at-risk pregnancies.

Our models were trained and validated using data specific to the state of Pernambuco, Brazil. This geographic and demographic specificity may limit the generalizability of our findings to other regions or populations with different healthcare conditions and risk factors. Although our methodological approach can be replicated elsewhere, the performance metrics we report might vary significantly when applied to data from other contexts.

## 6 Conclusions and next steps

In this work, we analyzed different data balancing techniques to evaluate machine learning models aimed at predicting preterm births and assessed their impact on machine learning performance. Each technique affects the solution of our problem in distinct ways, adapting to the specific needs of each case.

The results indicated that the insertion of synthetic data into the minority class improved the performance of models’ learning, particularly those based on Decision Trees. However, it is observed that the creation of synthetic data can be beneficial only up to a certain point. For example, in scenarios where the amount of original data in the minority class tripled, the models showed better overall performance than other balancing approaches.

The exclusive use of Undersampling and Oversampling techniques demonstrated significant limitations. Undersampling led to a substantial loss of information, while Oversampling resulted in bias towards the majority class. On the other hand, the hybrid sampling approach, which combines Undersampling and Oversampling, proved more efficient, especially in the scenario with tripled data from the minority class.

This study underscores the crucial role of sophisticated data balancing strategies in constructing reliable machine learning models for preterm birth prediction. Accurate predictions can significantly aid in early intervention strategies, potentially reducing the incidence of preterm births and improving neonatal health outcomes. The implications for public health policy are profound, suggesting that investments in data quality and machine learning capabilities could yield substantial benefits in maternal and child health sectors. Moreover, the novelty of dataset exploration, consideration of imbalance issues, and employment of machine learning models to predict preterm birth constitute the main contribution of this study, establishing the baseline results for future investigations using this data, which could propose more sophisticated methods to improve accuracy.

Moving forward, it would be advantageous to explore additional hybrid data balancing techniques and analyze their potential to generalize across different healthcare datasets. Further research should also investigate the scalability of these methods in larger, more diverse populations to enhance the robustness and applicability of predictive models. This progressive approach could impact healthcare delivery by enabling proactive management of pregnancy risks, thereby shaping better health policy and clinical practices.

We plan to explore external validation using other dataset (that may include medical images as well) and to conduct a more comprehensive comparison while taking these differences into account. And we also acknowledge that exploring more recent boosting algorithms, like XGBoost, CatBoost, and LightGBM, could potentially enhance models’ performance. These algorithms are designed to handle large, complex datasets more efficiently and often produce higher accuracy by employing advanced techniques such as regularization and optimized tree building. We plan to extend our work by incorporating these advanced models i n future research, with a comprehensive comparison to determine whether the improvements in accuracy outweigh any potential trade-offs in interpretability or computational cost.
